# The effect of reduced glutathione on the toxicity of silver diamine fluoride in rat pulpal cells

**DOI:** 10.1590/1678-7757-2020-0859

**Published:** 2021-04-19

**Authors:** Seunggun KIM, Mohannad NASSAR, Yukihiko TAMURA, Noriko HIRAISHI, Ahmed JAMLEH, Toru NIKAIDO, Junji TAGAMI

**Affiliations:** 1 Tokyo Medical and Dental University Graduate School of Medical and Dental Sciences Department of Cariology and Operative Dentistry Tokyo Japan Tokyo Medical and Dental University, Graduate School of Medical and Dental Sciences, Department of Cariology and Operative Dentistry, Tokyo, Japan.; 2 University of Sharjah College of Dental Medicine Department of Preventive and Restorative Dentistry Sharjah United Arab Emirates University of Sharjah, College of Dental Medicine, Department of Preventive and Restorative Dentistry, Sharjah, United Arab Emirates (UAE); 3 Tokyo Medical and Dental University Tokyo Japan Tokyo Medical and Dental University, Bio-Matrix (Dental Pharmacology), Tokyo, Japan.; 4 King Saud bin Abdulaziz University for Health Sciences College of Dentistry, Restorative and Prosthetic Dental Sciences Riyadh Saudi Arabia National Guard Health Affairs, King Saud bin Abdulaziz University for Health Sciences, College of Dentistry, Restorative and Prosthetic Dental Sciences, Riyadh, Saudi Arabia.; 5 King Abdullah International Medical Research Centre Riyadh Saudi Arabia National Guard Health Affairs, King Abdullah International Medical Research Centre, Riyadh, Saudi Arabia.; 6 Asahi University School of Dentistry Department of Operative Dentistry Gifu Japan Asahi University, School of Dentistry, Division of Oral Functional Science and Rehabilitation, Department of Operative Dentistry, Gifu, Japan.

**Keywords:** Cytotoxicity, Glutathione, Pulp cells, Silver diamine fluoride

## Abstract

**Introduction:**

Due to its ability to arrest untreated dental caries, silver diamine fluoride (SDF) has been advocated for indirect pulp capping procedures. However, the high concentrations of silver and fluoride in SDF raise concerns about its biocompatibility to pulpal tissues.

**Objectives:**

This study aimed to investigate the effect of SDF on the viability, alkaline phosphatase (ALP) activity, and morphology of pulpal-like cells (RPC-C2A) and to evaluate the influence of reduced glutathione (GSH) on SDF-induced cytotoxicity and deposit formation on dentin.

**Methodology:**

The cytotoxicity of diluted 38% SDF solutions (10-4 and 10-5), with or without the addition of 5 mM or 50 mM GSH, was evaluated at 6 and 24 hours. Cell viability was detected using WST-8 and the effect on ALP activity was performed using an ALP assay kit. Cell morphology was observed using a phase-contrast microscope. Scanning electron microscopy analysis was conducted to evaluate the effect of GSH incorporation or conditioning on SDF-induced deposit formation on dentin discs. Cytotoxicity data were analyzed by two-way analysis of variance (ANOVA) and Tukey post hoc tests (p<0.05).

**Results:**

There were significant differences between the groups. The results demonstrated that all tested SDF dilutions caused a remarkable cytotoxic effect, while the addition of GSH prevented SDF-induced damage at 6-hour exposure time in the higher dilution of SDF. Dentin treated with plain SDF or GSH-incorporated SDF solution showed deposit formation with occluded dentinal tubules, unlike the other groups.

**Conclusion:**

SDF severely disturbed the viability, mineralization-ability, and morphology of pulpal-like cells, while controlled concentrations of GSH had a short-term protective effect against SDF-induced damage. GSH showed an inhibitory effect on SDF-induced dentinal deposit formation. Further research is warranted to evaluate the effect of GSH on caries-arresting, anti-hypersensitivity, and antibacterial functions of SDF.

## Introduction

Silver diamine fluoride (SDF), an alkaline colorless solution composed of 24–29% silver (Ag) and 5–6% fluoride (F), is used for arresting caries progression and treating dentin hypersensitivity. This agent is gaining considerable attention due to the promising results of both clinical and laboratory studies.^1^ The mechanisms of action are still not clearly understood. However, it is believed that Ag ions provide antibacterial properties against some cariogenic bacteria, and F ions enhance remineralization.^2,3^ SDF also forms insoluble precipitates with calcium and phosphate that physically block dentinal tubules, minimizing dentin hypersensitivity.^4^ SDF application is considered a simple, inexpensive, and non-invasive procedure. Nevertheless, it can lead to discoloration of teeth.^5^ The Ag ions can infiltrate within demineralized and the underlying mineralized dentin.^6^ Ag and F ions can penetrate up to 0.2 mm into dentin.^7^ Despite the popularity of SDF, some reports suggest a cautious application of this solution into the oral cavity.^8,9^ Compared to silver chloride, SDF has higher toxicity against osteoblast-like cells and human gingival fibroblasts (HGF) — only one hour of contact with HGF induced irreversible cell death by necrosis.^8^ F concentration (44,800 ppm) in SDF is considered one of the highest compared to other available products.^10^ The high concentrations of Ag and F raise concerns about their harmful effects on pulpal tissue. The cytotoxic effect of SDF on HGF occurred even at low concentrations (0.1%) and lasted for nine weeks even after rinsing SDF-treated dentin discs with saline.^9^ Despite inadequate investigations on the cytotoxic effect of SDF on pulpal cells, studies show the potential use of SDF in indirect pulp capping procedure in deep cavities and gained much attention due to SDF’s ability to induce tertiary dentin formation.^11^

Glutathione is a low molecular weight thiol-compound that contains a sulfhydryl group in its structure. It has an essential role as an antioxidant that functions by various mechanisms such as metal chelators and radical quenchers.^12,13^ Glutathione is possibly the most prevalent and most important intracellular thiol-disulfide redox buffer in mammalian cells. The active form of glutathione is called reduced glutathione (GSH), a water-soluble tripeptide containing cysteine, glutamic acid, and glycine that contributes to the intracellular non-protein thiols.^14-16^ Ag cytotoxicity on rat and human liver cells could partially result from GSH depletion.^17,18^ The strong affinity of Ag to the sulfhydryl group could be responsible for the observed GSH depletion.^19^ Moreover, GSH is involved in the detoxification of Ag from blood plasma.^20^ F is also known to decrease the level of GSH, which reflects increased utilization of the latter due to the oxidative stress generated by the administration of F at a level of 25 ppm in the drinking water of rats.^21^ The increased oxidative stress and the adverse effect of F on the antioxidant function have also been confirmed in several other studies.^22-26^

There is a paucity of scholarly literature on the adverse effect of SDF on pulpal cells and preventing or minimizing this damage. Thus, this study aims to assess the effect of SDF on rat-pulpal cells, test the effect of GSH on SDF-induced damage on the cells as mentioned earlier, and its effect on the ability of SDF to form deposits on the surface of dentin discs. The tested null hypotheses were that: (i) SDF has no toxic effect on pulpal-like cells, (ii) GSH does not counteract the adverse effect of SDF on pulpal-like cells, and (iii) GSH does not affect SDF-induced deposit formation on dentin surfaces.

## Methodology

### Effect on cell proliferation, ALP activity, and morphology

#### Cell proliferation assay

The clonal cell line (RPC-C2A), pulp-like cells, was used in this study.^27^ To each well of 24-well culture plates, RPC-C2A cells (5_X_10^4^ cells/well) were placed and incubated for 24 h in a 5% CO_2_ incubator at 37°C. Six wells were allocated for each test solution for an observation time of 6 and 24 h. The test solutions included two dilutions of 38% SDF (1,000 and 10,000) (Bee Brand Medico Dental, Osaka, Japan) with or without 50 or 500 mM of GSH (L-Glutathione reduced, Sigma Aldrich Co., St. Louis, MO, USA). The prepared solutions were further diluted in a cell culture medium that contained 10% FBS in DMEM (Sigma-Aldrich, MO, USA), at a ratio of 1:9 (solution:medium).

Using a one-way ANOVA test, the sample size was calculated based on a significance level of 5% and power of 80% to detect a difference of 0.2 in spectrophotometric absorbance. The required sample size for each group is at least 4 (powerandsamplesize.com).

The resultant experimental solutions were as the following (n=6): (I) SDF_X_10^-4^ (pH 8.2), (II) SDF_X_10^-5^ (pH 8.11), (III) SDF_X_10^-4^+ 5 mM GSH (pH 7.6), (IV) SDF_X_10^-4^+ 50 mM GSH (pH 6.12), (V) SDF_X_10^-5^+ 5 mM GSH (pH 7.58), and (VI) SDF_X_10^-5^+ 50 mM GSH (pH 6.3). Because of the strong alkaline property of SDF, we diluted SDF to a concentration equal or lower than SDF_X_10^-4^ to have a pH less than 8.0, which caused no precipitation or turbidity in the cell culture medium. Cell culture in fresh medium (pH 7.74) without experimental solution served as the control. After the incubation time, the culture medium was discarded, and cells were washed with 200 µL phosphate buffer solution to prevent any interaction between the test solutions and the colorimetric assay. One hundred microliters of new culture medium were added to each well, and cell viability was measured using 2-(2-Methoxy-4-nitrophenyl)-3-(4-nitrophenyl)-5-(2,4-disulfophenyl)-2H-tetrazolium, mono-sodium salt [WST-8] (Cell Counting kit-8; Dojindo, Tokyo, Japan), which depends on the ability of mitochondrial dehydrogenases to oxidize WST-8 into a soluble, purple formazan. The spectrophotometric absorbance (optical density [OD]) of the samples at 450 nm was measured using a microplate reader. A blank well was regularly used for data subtraction by placing the same volume of culture medium with WST-8 solution into culture wells. The morphology of the cultured cells was observed by using a phase-contrast microscope (1X70; Olympus, Tokyo, Japan).

## ALP activity measurement

Cultured RPC-C2A cells were treated with the same solutions described above for 6 or 24 h (n=6). ALP activity was determined by using ALP Assay Kit (Takara Bio, Shiga, Japan).

## Scanning electron microscopy analysis of dentin discs treated with SDF

According to the protocol approved by the Human Research Ethics Committee, extracted human non-carious third molars were used in this part of the study. Flat dentin discs were created perpendicular to the tooth’s longitudinal axis, using a slow-speed diamond saw (Isomet Low Speed Saw; Buehler, Lake Bluff, IL, USA) under water lubrication. Dentin surfaces were polished using up to 4000-grit silicon-carbide paper under water irrigation, followed by sonication with deionized water for debris removal. The dentin discs were randomly divided into four groups. In group 1 (control group), dentin discs were treated with distilled water. In groups 2 and 3, 38% SDF (pH 10) was applied, followed by either distilled water or 20% GSH solution (pH 4), respectively. Group 4 discs were treated with a solution that contained 38% SDF and 20% GSH (pH 6.8-7.8). All solutions were applied with agitation using micro-brushes for 1 min. A final rinse with water for 30 s was performed on all dentin discs. Specimens were stored in a simulated body fluid (SBF) of a 7.4 pH for 6 h. SBF solution was prepared by dissolving 136.8 mM NaCl, 4.2 mM NHCO_3_, 3 mM KCl, 1mM K_2_HPO_4_·٣H_2_O, 1.5 mM MgCl_2_ and 0.5 mM Na_2_SO_4_ in water.^28^ Specimens were dried inside a covered glass vial, sputter-coated with gold/palladium, and then observed under a scanning electron microscope (SEM) (JSM-IT100 scanning microscope; JEOL, Tokyo, Japan) operating at 20 kV.

## Statistical Analysis

Statistical analysis was performed by applying a two-way analysis of variance (ANOVA) and Tukey’s post hoc test using the experimental solution and exposure time as two factors. The analysis was performed using SPSS (IBM SPSS Statistics, v21; IBM Corp.) with a significance level set at 5%.

## Results

### Effect on viability, ALP activity, and morphology of RPC-C2A cells

**Figure 1:**
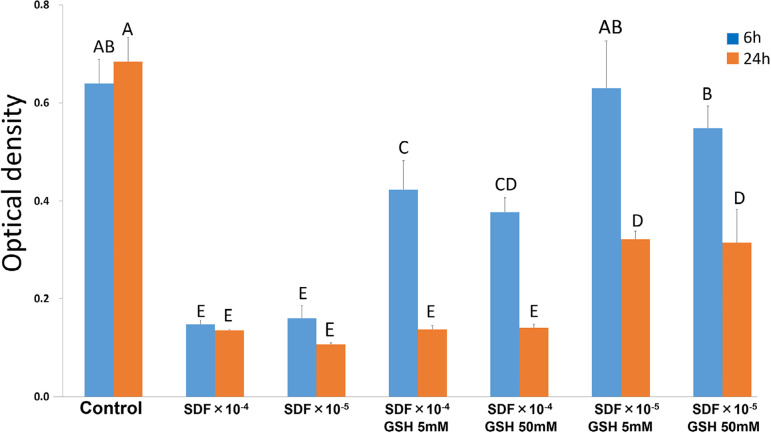
Cytotoxicity of culture medium on RPC-C2A cells containing diluted SDF with and without GSH after 6 and 24 h of incubation. Cell viability was determined by using WST-8 assay. Data were analysed by using two-way ANOVA. The same letter indicates no significant differ-ence (P>.05)

Figure 1 shows the effect of the tested solutions on the viability of RPC-C2A cells after 6 or 24 h of exposure. At both exposure times, the two dilutions of SDF showed lower OD values than the control group (p<0.001). At 6 h, SDF_X_10^-4^+ 5 mM GSH and SDF_X_10^-4^+ 50 mM GSH solutions had OD values that were statistically higher than both dilutions of SDF without GSH (p<0.001); however, these values were statistically significantly lower than the control group (p<0.001). Meanwhile, there was no statistically significant difference among SDF_X_10^-5^, SDF_X_10^-4^, SDF_X_10^-4^+ 5 mM GSH and SDF_X_10^-4^+ 50 mM GSH at 24 h (p=0.628). At both exposure times, the OD values obtained in SDF_X_10^-5^+ 5 mM GSH and SDF_X_10^-5^+ 50 mM GSH solutions were statistically higher than both dilutions of SDF without GSH (p<0.001). At 6 h, SDF_X_10^-5^+ 5 mM GSH and SDF_X_10^-5^+ 50 mM GSH solutions had OD values that were comparable to the control group (p=0.113); however, at 24 h, the resultant OD values were statistically significantly lower than the control group(p<0.001). At both exposure times, SDF_X_10^-5^+ 5 mM GSH and SDF_X_10^-5^+ 50 mM GSH solutions had OD values that were statistically significantly higher than all other experimental solutions of the same exposure time (p<0.001).

**Figure 2:**
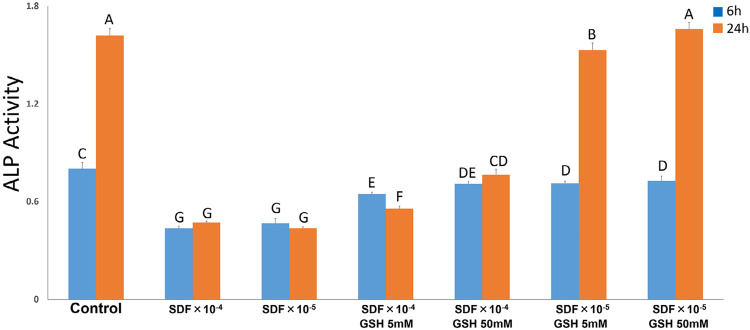
ALP activity of RPC-C2A cells cultured with diluted of SDF with and without GSH for 6 and 24 h. Data were analysed by using two-way ANOVA. letter indicates no significant differ-ence (P>.05)

Figure 2 shows the effect of the tested solutions on the ALP activity of RPC-C2A cells after 6 or 24 h of exposure. At both exposure times, the two plain dilutions of SDF showed statistically significantly lower ALP activity than the control group (p<0.001). All solutions containing GSH had ALP activities that were statistically significantly higher than the two plain dilutions of SDF, at both exposure times (p<0.001). At 6-h exposure time, all solutions containing GSH had ALP activities that were lower than the control (p<0.001), while at 24-h exposure time, SDF_X_10^-5^+ 50 mM GSH group showed an ALP activity that is comparable to the control (p=0.727).

**Figure 3:**
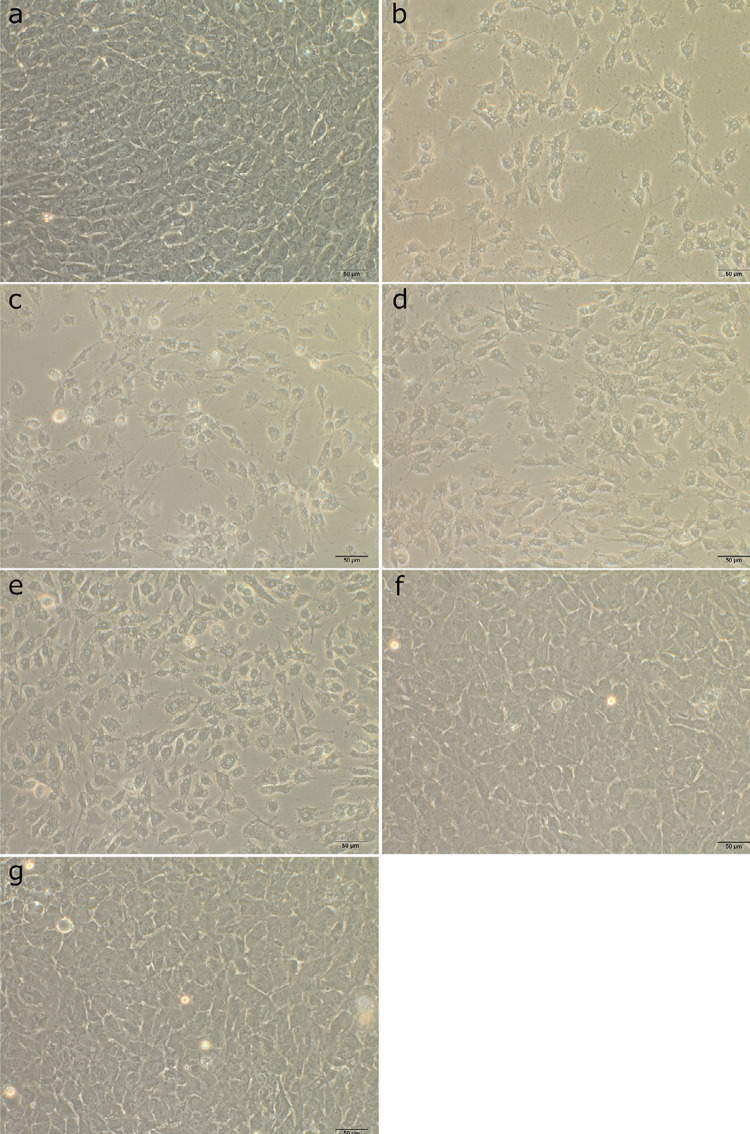
Morphologic changes of RPC-C2A cells after 24 h of exposure to test solutions. (a) Polygonal-shaped cells of the control group. (b-e) Cells treated with SDFX10-4, SDFX10-5, SDFX10-4+5 mM GSH, or SDFX10-4+50 mM GSH, respectively, showed contracted and spheri-cal morphology and increases in intercellular spaces were observed. (f and g) Cells treated with SDFX10-5+5 mM GSH or SDFX10-5+50 mM GSH, respectively, exhibited mostly normal polygo-nal morphology

Figure 3 shows cell morphology at 24 h of all groups. Morphologically, RPC-C2A cells in the control media showed polygonal appearance (Figure 3a), whereas cells treated with SDF_X_10^-4^(Figure 3b) or SDF_X_10^-5^(Figure 3c) exhibited contracted and spherical morphology with increased intercellular spaces suggestive of cellular death and decreased proliferation. At 6 h, most of the cells in the GSH-containing groups exhibited normal polygonal morphology (data not shown); however, at 24 h, decreased cellular density and increased intercellular spaces for both GSH concentrations with SDFx10^-4^ (Figure 3d and e) were observed while these changes were less evident in the groups containing GSH with SDFx10^-5^(Figure 3f and g) where most of the cells showed normal morphology.

### SEM analysis of dentin discs treated with SDF

**Figure 4:**
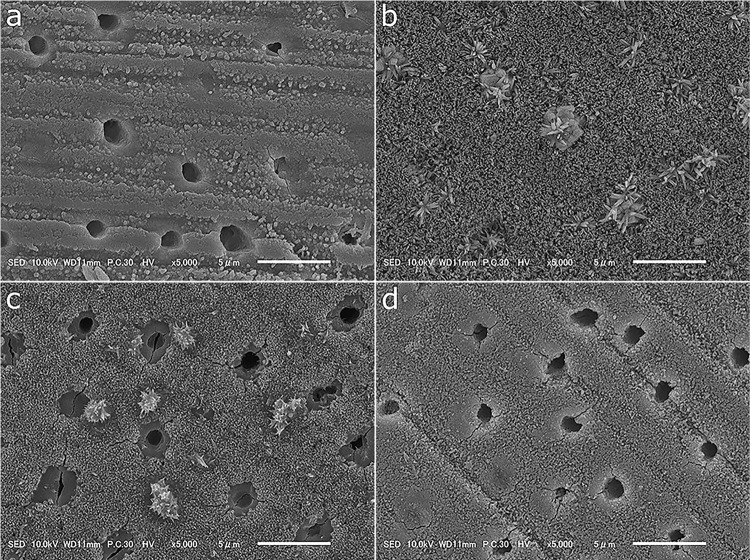
Topographical scanning electron microscopy images of flat dentinal surfaces treated with different solutions. (a) control, (b) 38% SDF followed by distilled water, (c) 38% SDF incor-porated with 20% GSH followed by distilled water, and (d) 38% SDF followed by 20% GSH ap-plication

Dentin discs treated with 38% SDF followed by a rinse with distilled water showed marked mineral deposit formation on the surface along with occluded dentinal tubules (Figure 4b). Nevertheless, these findings were less prominent on dentinal surfaces treated with the solution that contained 38% SDF and 20% GSH, followed by a rinse with distilled water (Figure 4c). Meanwhile, dentin discs treated with 38% SDF followed by conditioning with 20% GSH showed less deposit formation than the aforementioned experimental groups (Figure 4d). The control group showed the least amount of deposit formation (Figure 4a).

## Discussion

This study is the first to assess the effect of SDF on pulpal-like cells and the efficacy of GSH to mitigate SDF-induced toxicity. We rejected the null hypotheses because SDF adversely affected the viability, ALP activity and morphology of the used cells, and GSH at specific concentrations and exposure time mitigated SDF-induced toxicity and negatively affected SDF-induced deposit formation on dentin.

One of the recently suggested SDF applications is its use in deep cavities for indirect pulp capping procedures due to the claimed ability of SDF to induce tertiary dentin formation.^11^ Despite the established safety of SDF application in this procedure, there is always a concern about the effect of the released F and Ag ions^8,9,^ which can penetrate easily into dentin, especially within demineralized dentin left at the deepest part of the cavity preparation in indirect pulp procedures,^6,7^ thus the need for a non-toxic indirect pulp capping agent cannot be stressed enough. Due to the chances of pulpal cells exposure to SDF, pulpal-like cells were used in this study to assess its effect on the viability, ALP activity, and morphology of these cells. In a preliminary study, we used higher SDF concentrations in the cell culture medium than the concentrations used in this study. We noticed white turbidity and increased pH (>8) of cell culture medium at high SDF concentrations. Thus, in this study, 38% SDF was diluted at 10,000-fold (0.0038%) and 100,000-fold (0.00038%), which showed no turbidity in the culture medium. SDF at 0.0038% and 0.00038% adversely affected cell viability and changed cellular morphology from polygonal to round with loss of intercellular spaces. SDF also suppressed the ALP activity of the cells. ALP activity correlates with the mineralization ability of the cells.^29^ Thus, it is essential to maintain this activity for successful indirect pulp therapy procedure by the formation of tertiary dentin. It was reported that the cytotoxicity of SDF is related to both F and Ag constituents with a synergistic interaction between these two constituents that heightens the cellular damage^8^ and enhances oxidative stress.^30^ The mechanism of this enhanced toxic effect of Ag and F is speculated to depend on the increased generation of reactive oxygen species (ROS) or lipid peroxidation accompanied by a reduction in the total antioxidant capacity leading to cell death and inflammation.^30^ In this study, the supplementation of the experimental solutions with 5 mM or 50 mM GSH led to a decreased toxic effect of both dilutions of SDF at 6-h exposure time; however, this protective effect was lost at 24-h exposure time for both GSH concentrations with 0.0038% SDF. This confirms the finding that SDF has a long-standing effect even at low concentration, which is beneficial for its antimicrobial application; nonetheless, it also raises concerns about its cytotoxicity.^9^ This prolonged effect is referred to as the “zombie effect”, where dead cells act as a reservoir of Ag, further affecting healthy nearby cells.^31^ As stated earlier, both Ag and F cause depletion in the level of GSH,^19,21^ thus, several mechanisms can be proposed for the action of GSH in protecting pulpal cells from SDF-induced damage: (i) enhancement of cellular levels of GSH,^32^ (ii) reduction of ROS levels;^33^ and/or (iii) direct interaction of GSH with Ag resulting in the formation of complexes that help in detoxification of Ag.^20^

We assessed the effect of GSH on SDF-induced deposit formation , and the results showed that applying 20% GSH as a separate step after SDF significantly decreased the formation of these deposits with several open dentinal tubules. However, when GSH was incorporated within SDF, this inhibitory effect on deposit formation was less pronounced. GSH was previously studied for its ability to decrease SDF-induced color changes of tooth structure.^34^ The process through which GSH affects the function of SDF is still unclear; however, the interaction with Ag and/or the acidity of GSH might have contributed to the current findings. The demineralizing effect of a low pH agent was suggested to harm the stability of crystal precipitations on dentin surfaces,^35^ and this might be true for the group where 20% GSH was applied separately on dentin after SDF application. Both F and Ag ions contribute to the formation of SDF-induced precipitates.^36,37^ Significant Ag layer accumulation is reported on dentin surfaces treated with SDF.^38^ GSH is known to interact with silver,^19,20,39^ and we speculate that the formation of these complexes could minimize the action of Ag in forming crystals on dentin surfaces.

## Conclusion

The limitations of this study include, but are not limited to, the utilization of extracted human teeth from different subjects and the use of *in vitro* experimental conditions that contribute to only limited answers to more complex problems. Within these limitations, SDF showed detrimental effects on the viability, ALP activity, and morphology of the pulpal-like cells. The use of GSH at specific concentrations and exposure times attenuated the toxic effect of SDF; however, it negatively affected SDF-induced deposit formation on dentin. This protective effect of GSH may provide insights into the development of a “smart,” more biocompatible agent. Further research could assess the repercussions of GSH application on caries-arresting, anti-hypersensitivity, and antibacterial functions of SDF using *in vivo* animal models.
